# Mild Transient Hypercapnia as a Novel Fear Conditioning Stimulus Allowing Re-Exposure during Sleep

**DOI:** 10.1371/journal.pone.0067435

**Published:** 2013-06-26

**Authors:** Angela L. McDowell, Ashlee B. Filippone, Alex Balbir, Anne Germain, Christopher P. O’Donnell

**Affiliations:** 1 Department of Medicine, University of Pittsburgh, Pittsburgh, Pennsylvania, United States of America; 2 Department of Psychiatry, University of Pittsburgh, Pittsburgh, Pennsylvania, United States of America; McLean Hospital/Harvard Medical School, United States of America

## Abstract

**Introduction:**

Studies suggest that sleep plays a role in traumatic memories and that treatment of sleep disorders may help alleviate symptoms of posttraumatic stress disorder. Fear-conditioning paradigms in rodents are used to investigate causal mechanisms of fear acquisition and the relationship between sleep and posttraumatic behaviors. We developed a novel conditioning stimulus (CS) that evoked fear and was subsequently used to study re-exposure to the CS during sleep.

**Methods:**

Experiment 1 assessed physiological responses to a conditioned stimulus (mild transient hypercapnia, mtHC; 3.0% CO_2_; n = 17)+footshock for the purpose of establishing a novel CS in male FVB/J mice. Responses to the novel CS were compared to tone+footshock (n = 18) and control groups of tone alone (n = 17) and mild transient hypercapnia alone (n = 10). A second proof of principle experiment re-exposed animals during sleep to mild transient hypercapnia or air (control) to study sleep processes related to the CS.

**Results:**

Footshock elicited a response of acute tachycardia (30–40 bpm) and increased plasma epinephrine. When tone predicted footshock it elicited mild hypertension (1–2 mmHg) and a three-fold increase in plasma epinephrine. When mtHC predicted footshock it also induced mild hypertension, but additionally elicited a conditioned bradycardia and a smaller increase in plasma epinephrine. The overall mean 24 hour sleep–wake profile was unaffected immediately after fear conditioning.

**Discussion:**

Our study demonstrates the efficacy of mtHC as a conditioning stimulus that is perceptible but innocuous (relative to tone) and applicable during sleep. This novel model will allow future studies to explore sleep-dependent mechanisms underlying maladaptive fear responses, as well as elucidate the moderators of the relationship between fear responses and sleep.

## Introduction

An emerging literature suggests that posttraumatic stress disorder (PTSD) and sleep are intimately linked in a bi-directional relationship – PTSD compromises normal sleep, which increases the risk of and exacerbates the magnitude of PTSD [1]–[5]. Sleep disturbances occurring after exposure to traumatic events increase the risk for developing PTSD [6], [7], whereas treatment of sleep disturbances alleviates those symptoms [8]–[10]. The obvious ethical concerns associated with exposure or re-exposure of participants to harmful or threatening stimuli limit the extent that human studies can adequately determine a causal relationship between sleep disruption and maladaptive stress responses, making animal models important for investigating the underlying mechanistic links between sleep and fear responses. Animal studies have utilized fear-conditioning (FC) paradigms to gain insight into a variety of outcomes including fear acquisition and extinction and their relationship to sleep [11]–[16] as well as to model components of human PTSD [17], [18].

Classical FC involves the temporal pairing of an initially innocuous stimulus (e.g. auditory tone; CS) with a biologically salient stimulus (e.g. footshock; unconditioned stimulus, US) that elicits a reflexive response (unconditioned response). Through a single optimal or repeated pairing(s) the CS will ultimately elicit similar behavioral and physiological responses as the UCS (conditioned response). To date, animal studies have investigated the effects of sleep disruption on learning of a FC response [19]–[21] or on the impact of re-exposure to a CS during wakefulness on subsequent sleep patterns [16], [22], [23]. However, the effects of re-exposure *during* sleep to an acquired CS have only been explored using an aural cue at an altered and restricted duration from the initial pairing [24]. The primary reason for a lack of studies investigating sleep-related fear responses to specific cues is that conditioned stimuli are typically arousing (e.g. tone, light) and can elicit a startle response [12] which could awaken the subject from sleep (for review see [25]). To address the issue of re-administering a CS during sleep, it is necessary to develop a model that incorporates delivery of a perceptible, yet innocuous and minimally or non-arousing stimulus within the framework of an automated sleep detection system.

In previous work we have observed that mice can be exposed to hypercapnia during sleep without inducing arousal [26]–[28]. Therefore, we proposed to utilize mild (3% inspired CO_2_), transient (60 sec) hypercapnia (mtHC) as a CS that could subsequently be used for re-exposure during sleep. Moreover, we have previously developed an algorithm based real-time sleep scoring system [29] that was adapted to automatically trigger delivery of a CS of mtHC specifically during sleep. Thus, the purpose of our study was two-fold. In Experiment 1, we compared physiological responses utilizing the novel FC paradigm of mtHC-footshock with the commonly used tone-footshock FC paradigm with the goal of establishing mtHC as an acceptable CS. We hypothesized that repetitive pairings of mtHC-footshock would produce acquisition of learned physiologic fear responses. In Experiment 2, we conducted a pilot study to demonstrate proof of principle that mtHC could be successfully re-administered during sleep to previously fear conditioned animals.

## Methods

### Animals

Experiments were conducted in adult male FVB/J mice at 10–12 weeks old from Jackson Laboratories (Bar Harbor, ME). Animals were maintained on a 12∶12 hour light-dark cycle and given seven days of adaptation prior to recording. Animals were housed in a customized pyramidal chamber [7″ (W) x 9″ (H) x 7″ (L)] with continuous access to food and water that was designed for delivery of gas (entered through inlet ports in the base and exhausted through an open hole at the apex). The bottom of the chamber was removable and replaced by an electric grid (H10-11M-TC; Coulbourn) to induce footshock. The chamber was contained inside a light-controlled and sound-dampening chamber [22″ (L) x 16.5″ (H) x 14″ (W)]. All animals were housed in the same customized chambers throughout the entire adaptation and experimental period to control for environmental exposure. Animal handling and experimentation was conducted ethically and in accordance with approved Institutional Animal Care and Use Committee (IACUC) protocols at the University of Pittsburgh, as well as the Animal Care and Use Review Office (ACURO) of Department of the Army.

### Surgical Instrumentation

#### EEG and EMG instrumentation

**Figure 1:**
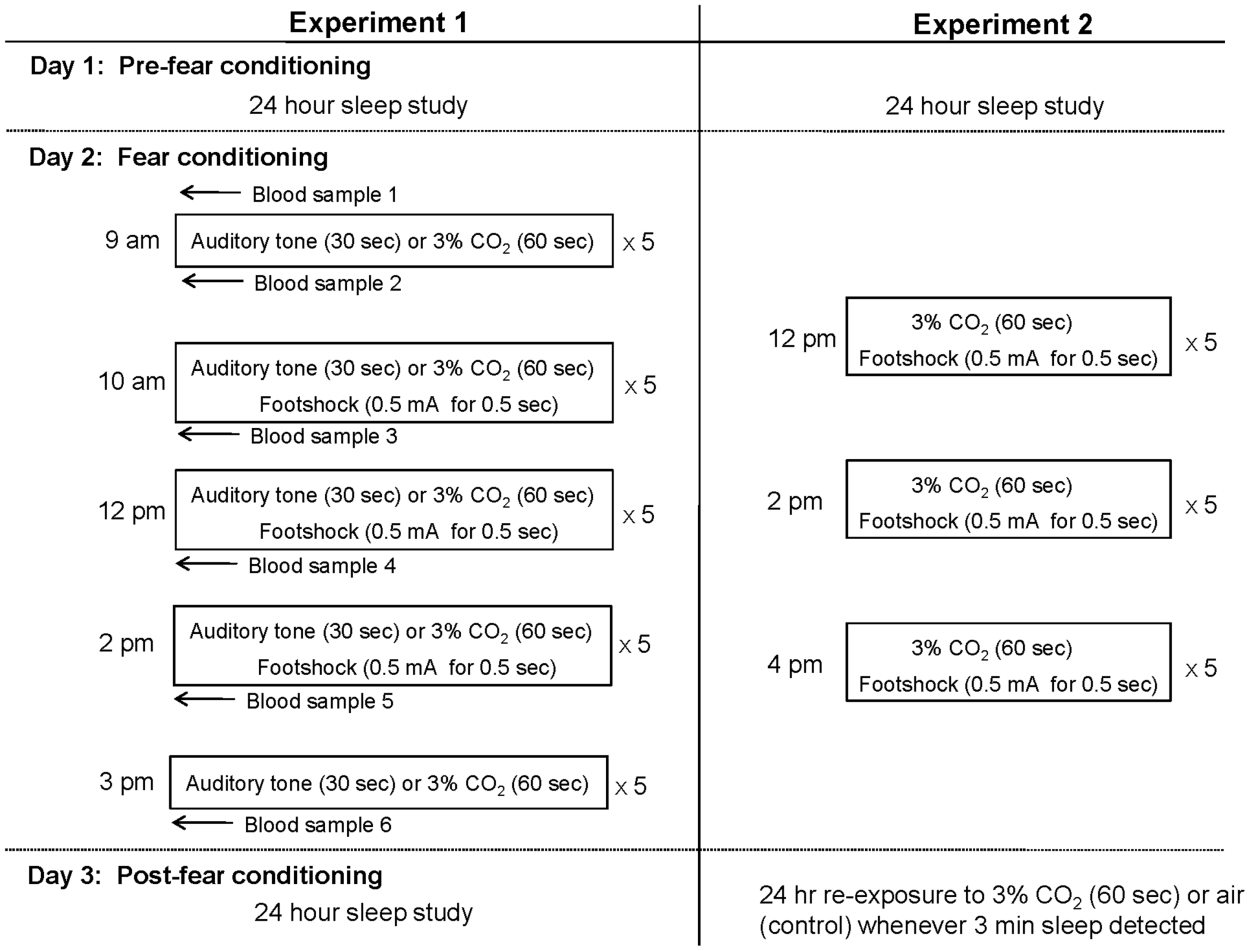
Shows the three day protocols for each fear-conditioning paradigm. In Experiment 1 baseline sleep (24 hours) data were collected prior to fear conditioning. Subsequently, animals were exposed to either CS alone (tone or 3% CO_2_) or CS+US exposures for five repeated series across five time points. CS alone exposures occurred at 9 am and 3 pm, while conditioning trials occurred at 10 am, 12 pm, and 2 pm. An arterial basal blood sample was taken before the first CS exposure and immediately following each of the five series of exposures. The right side shows the three day protocol for Experiment 2. Baseline sleep (24 hours) data were collected prior to fear conditioning. On the subsequent day animals were exposed to paired trials at 12 pm, 2 pm, and 4 pm. One hour following fear conditioning (5 pm), animals began 24 hours of re-exposure to the CS+ (mtHC) or CS− (air) for 60 sec whenever three minutes of consolidated sleep occurred.

Animals were anesthetized using 1 to 2% isoflurane for all surgical procedures and in effort to minimize suffering animals were monitored twice daily post-operatively and given pain medicine (0.3 mg/ml Buprenorphine) for three subsequent days. Electroencephalographic (EEG; E363/1, Plastics One, Roanoke, VA) electrodes and nuchal electromyographic (EMG; E363/76, Plastics One) electrodes were implanted as previously described [30]. A midline incision was made to expose the skull and muscles immediately posterior to the skull. The underlying fascia was gently cleared from the skull surface, three small burr holes were drilled through the skull in the left frontal and parietal regions and three EEG electrodes were fastened via jewel screws (diameter of 1.6 mm). The first electrode was placed 2–3 mm caudal to bregma and 1–2 mm lateral of the midsagitaal suture. The second electrode was placed 2–3 mm rostral to bregma and 1–2 mm lateral of the midsagitaal suture. The third electrode was placed 2–3 mm rostral to bregma and 1–2 mm lateral of the midsagitaal suture. Two nuchal EMG electrodes were stitched flat onto the surface of the muscle. In animals used in Experiment 2 (see figure 1) and in the mtHC alone group an EKG electrode was implanted subcutaneously and sutured onto the muscle overlying the area of the sixth rib and tunneled subcutaneously towards the head. The EEG, EMG and EKG electrodes were inserted into a pedestal (MS363, Plastics One) and secured to the skull with dental acrylic.

#### Arterial catheterization

In anesthetized mice a femoral artery catheter was chronically implanted as previously described [31]. The catheter was inserted in the left femoral artery, sutured in place, stabilized with superglue (Henkel Corp, Rocky Hill, CT, USA), tunneled subcutaneously to the upper back by threading through a blunt needle. The catheter was taped to a wire sutured to posterior cervical muscles for line security (792500; A-M-Systems, Sequim, WA, USA), and connected to a 360° swivel designed for mice (375/D/22QM; Instech, Plymouth Meeting, PA, USA) that worked in combination with the mercury swivel used to record polysomnography. Patency of the catheters was maintained by continuously flushing 7 µl hr^−1^ saline containing 20 U ml^−1^ heparin (Baxter, Deerfield, IL, USA) using a multi-syringe pump adaptor (R99-EM; Razel Scientific Instruments, St. Albans, VT, USA). Arterial blood pressure measurements were collected with pressure transducers (Cobe Inc.; Lakewood, CO) zeroed at mid-thoracic level. Calibrations were checked at the beginning and end of each experiment.

Animals were given five days of recovery before being tethered to the electrical and fluid swivels where they were given two additional days to adapt before baseline recordings were initiated. At time of tethering a connector cable from the animal was fixed above to a low friction mercury swivel allowing 360 degree unrestricted movement of the tethered mouse.

### Stimuli Presentation and Data Acquisition

For tone production, a Tone/Noise Generator (model A69-20) from Coulbourn Instruments (Whitehall, PA) was used to deliver a 2400 Hz, 80 dB tone. A Radio Shack Digital Sound Level meter was used to regulate the distance from the tone generator to the animal to achieve the required 2400 Hz and 80 dB stimulus. The mtHC stimulus of one minute of 3% CO_2_ was delivered through a compressed CO_2_ tank connected via tubing to three inlet ports on the base of the chamber. Gas levels were monitored via a CO_2_ analyzer (model 17625, Vacumed) also connected via inlets to the housing chamber. Electric footshock was produced with a Precision Regulated Animal Shocker with an electric floor shock grid (model H13-15) from Coulbourn Instruments (Whitehall, PA).

A Grass Instruments amplifier (Quincy, MA) was used to collect EEG activity (filtered 0.1–30 Hz), EMG activity (filtered 10–100 Hz), EKG and pulsatile arterial pressure. Signals from the Grass recorder were collected using Windaq Pro acquisition software (Dataq Instruments; Akron, OH), were digitized at 300 Hz (DI-720 data acquisition board; Dataq Instruments; Akron, OH) and stored on optical disk.

### Procedure

#### Experiment 1

**Figure 2:**
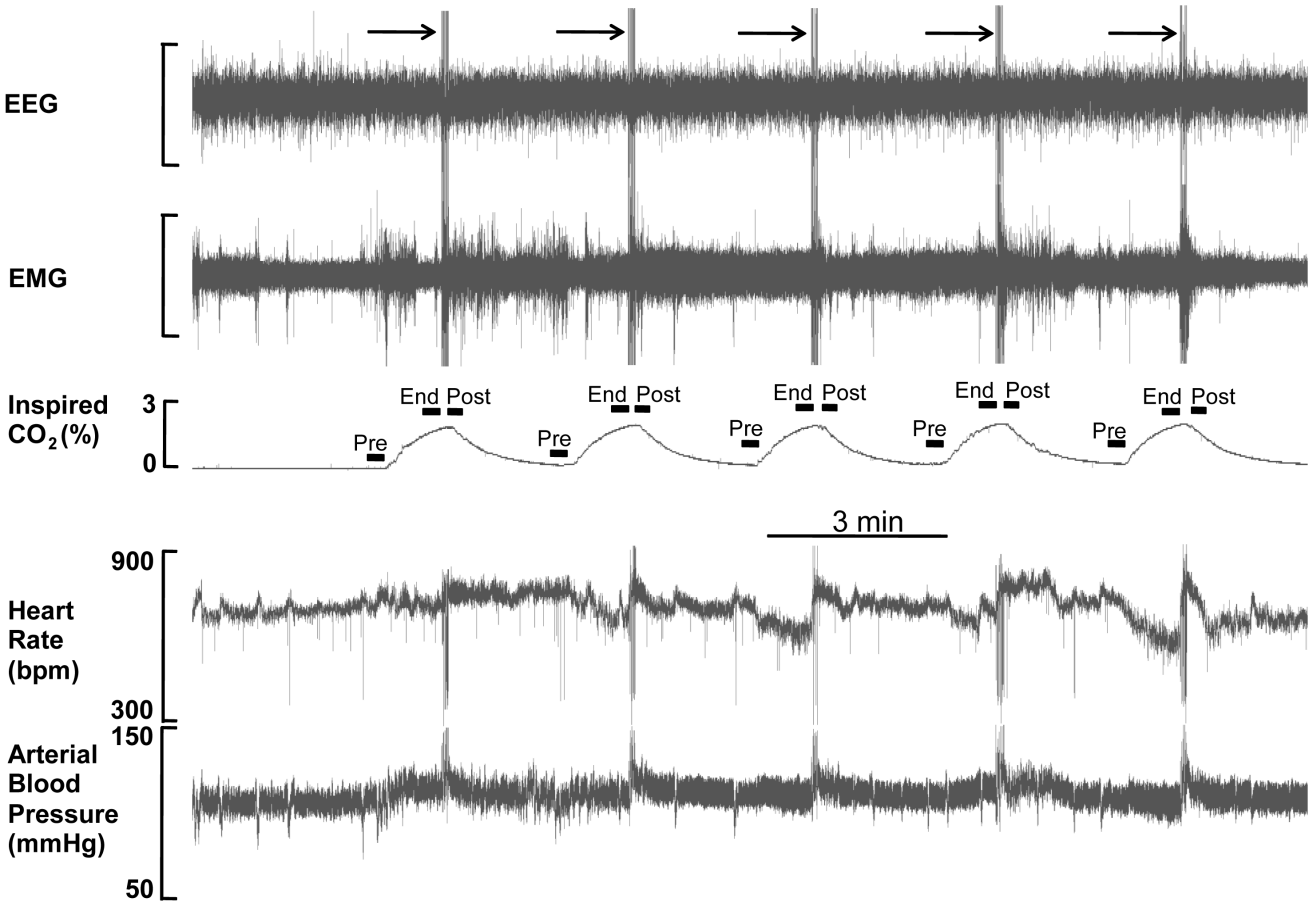
Shows an electroencephalographic (EEG), electromyographic (EMG), inspired CO_2_, heart rate, and arterial blood pressure tracing during one series of five one minute exposures to CO_2_ with each exposure followed by five footshock pulses. Shock-induced electrical artifact is evident in EEG and EMG tracings (top two traces marked by horizontal arrow). Heart rate and blood pressure were analyzed for the 10 sec prior to initiation of the conditioning stimulus (Pre), for the 10 sec immediately prior to footshock (End) and the 10 sec immediately after footshock (Post), and are marked by the short horizontal bars on the inspired CO_2_ tracing. Note the presence of bradycardia during exposure to each episode of mtHC (the transient artifact in the heart rate tracing at time of foot shock did not affect the determination of End and Post heart rate and blood pressure values).

On Day 1 a 24-hour baseline assessment of sleep was conducted and on Day 2 the animals underwent the fear conditioning protocol (Figure 1). Immediately following cessation of the protocol another 24-hour sleep assessment was conducted (Day 3). The fear-conditioning protocol involved five series of either paired (CS-US) or unpaired (CS alone) exposures at: 9 am (CS only), 10 am, 12 pm, 2 pm (CS-US), and 3 pm (CS only; Figure 1 and see sample tracing in Figure 2). Within each series, exposures were presented in 3 min. intervals. Four groups of animals were studied using two types of CS (tone or mtHC) in either the presence (T+FS, n = 18; mtHC+FS, n = 17) or absence (T, n = 17; mtHC, n = 10) of the US (footshock). One 30 second tone or one 60 second mtHC presentation predicted the onset of five footshock pulses (0.5 mA for 0.5 sec), which coincided with the offset of the CS. In total, there were five CS exposures for each time series listed above and each exposure was separated by three minute intervals. Each series (time point) consisted of five stimulus-footshock exposures for a total of 15 paired exposures and 10 unpaired exposures for the paired groups and 25 unpaired exposures for the CS only groups.

Also, six 40 ul blood samples were taken during the course of the fear-conditioning day for each animal in Experiment 1 (Figure 1). The first blood sample (basal) was taken at 8∶30 am prior to beginning the fear-conditioning paradigm. The other blood samples were taken immediately (<2 min) after the completion of each of the five series of exposures at 9 am, 10 am, 12 pm, 2 pm, and 3 pm. At the time of collection, blood samples were centrifuged and plasma samples were stored and frozen at −80°C for subsequent analyses. The separated red blood cells were mixed with 20 ul of 100U heparin solution until homogenous and re-infused back into the mouse to maintain circulating blood volume. Plasma epinephrine was measured using an ELISA assay kit (Rocky Mountain Diagnostics, Inc., Colorado Springs, CO).

#### Experiment 2

A separate group of animals (n = 6) were instrumented with EEG, EMG, and EKG electrodes and exposed to mtHC and footshock at 12 pm, 2 pm, and 4 pm in an identical manner to the CS-US pairing described above (note: there was no exposure to the CS alone; see Figure 1). Pre- and post-fear conditioning sleep data were collected for 24 hours and animals were re-exposed to either 60 sec of mtHC (n = 3) or air (n = 3) whenever three minutes of continuous sleep was recorded in the 24 hour post-fear conditioning period.

A computer-controlled automated sleep/wake detection system was implemented to control the delivery of mtHC to animals during sleep as previously described [29] and detailed below. Baseline data was collected and analyzed prior to stimulus re-exposure to determine the optimal threshold settings for each animal for detection of wake, NREM, and REM sleep. Once the thresholds were determined they were held constant throughout the 24 hour period of re-exposure to mtHC. A constant flow of room air at 5 l/min was delivered continuously through the base of the pyramidal chamber. Whenever three minutes of contiguous sleep (either NREM or REM sleep) was detected, the computer turned the gas state from off to on to deliver mtHC at 5 l/min for 60 sec. After 60 sec of exposure room air was again delivered to the chamber until the next period of 3 contiguous minutes of sleep was detected. An additional three animals that were similarly trained with three series of exposures to mtHC and footshock were re-exposed to a 60 sec period of room air at 5 l/min as a control for animals exposed to mtHC during sleep to account for any non-specific effects of gas flow changes.

### Analyses

#### Heart rate and blood pressure

Mean arterial blood pressure was derived from the pulsatile arterial blood pressure tracing and heart rate derived from either the blood pressure tracing (Experiment 1) or the interbeat interval from the EKG tracing (Experiment 2 and from some animals in Experiment 1 with insufficient pulse pressure to accurately derive heart rate). Mean arterial pressure and heart rate were measured at three time intervals for each individual exposure of the CS or paired CS-US. The heart rate and arterial pressure were averaged at three time points within each exposure: (1) Pre-stimulus (10 sec immediately prior to onset of the CS) (2) End-stimulus (10 sec at the end of the CS) and (3) Post-stimulus (10 sec directly after the termination of footshock exposure; see marked horizontal bars on CO_2_ tracing in Figure 2). In each animal the pre-, end-, and post-stimulus heart rates and blood pressures were averaged for each series of 9 am, 10 am, 12 pm, 2 pm, and 3 pm for Experiment 1. For Experiment 2, heart rate was averaged similarly at 12 pm, 2 pm, and 4 pm.

#### Sleep scoring

Sleep data were analyzed using a customized program that converted DATAQ digitized data files into Stanford Sleep Structure Scoring System (SSSSS) format for characterization of signals using the rodent software developed by Joel H. Benington [32] and subsequently validated in mice by Veasey and colleagues [33]. The program utilizes Fourier spectral analysis of the EEG in the delta (0.5–4.0 Hz), sigma (10.0–14.0 Hz), and theta (6.0–9.0 Hz) frequency bands in combination with the moving average of the EMG amplitude to determine sleep in 10 sec epochs. Twenty-four hour periods of data were plotted as sigma*theta power against EMG, and thresholds for the slope and intercept of the relationship were used to distinguish between sleep and wake. A second plot of the delta/theta power against EMG was used to distinguish non-rapid-eye movement (NREM) sleep from rapid eye movement (REM) sleep on the basis of a delta/theta threshold.

#### Statistics

All results are presented as means ± standard error of the mean (SEM). Statistical differences over time within an experimental group were determined by one-way ANOVA with repeated measures and statistical differences between groups were determined by two-way ANOVA. When the ANOVA was significant, statistical differences between means were determined by Dunnett’s post-hoc analyses to determine changes across time compared to baseline within an experimental group or by Tukey’s post-hoc analyses to determine differences between experimental groups. Heart rate and blood pressure differences between pre-stimulus to end-stimulus and end-stimulus to post-stimulus were tested for statistical significance using a two-tailed, paired Student t-test. Due to insufficient power, statistical tests were not performed for the proof of principle pilot study (Experiment 2) comparing the mtHC and air (control) groups containing three animals each.

## Results

### Experiment 1

#### Heart rate

**Figure 3:**
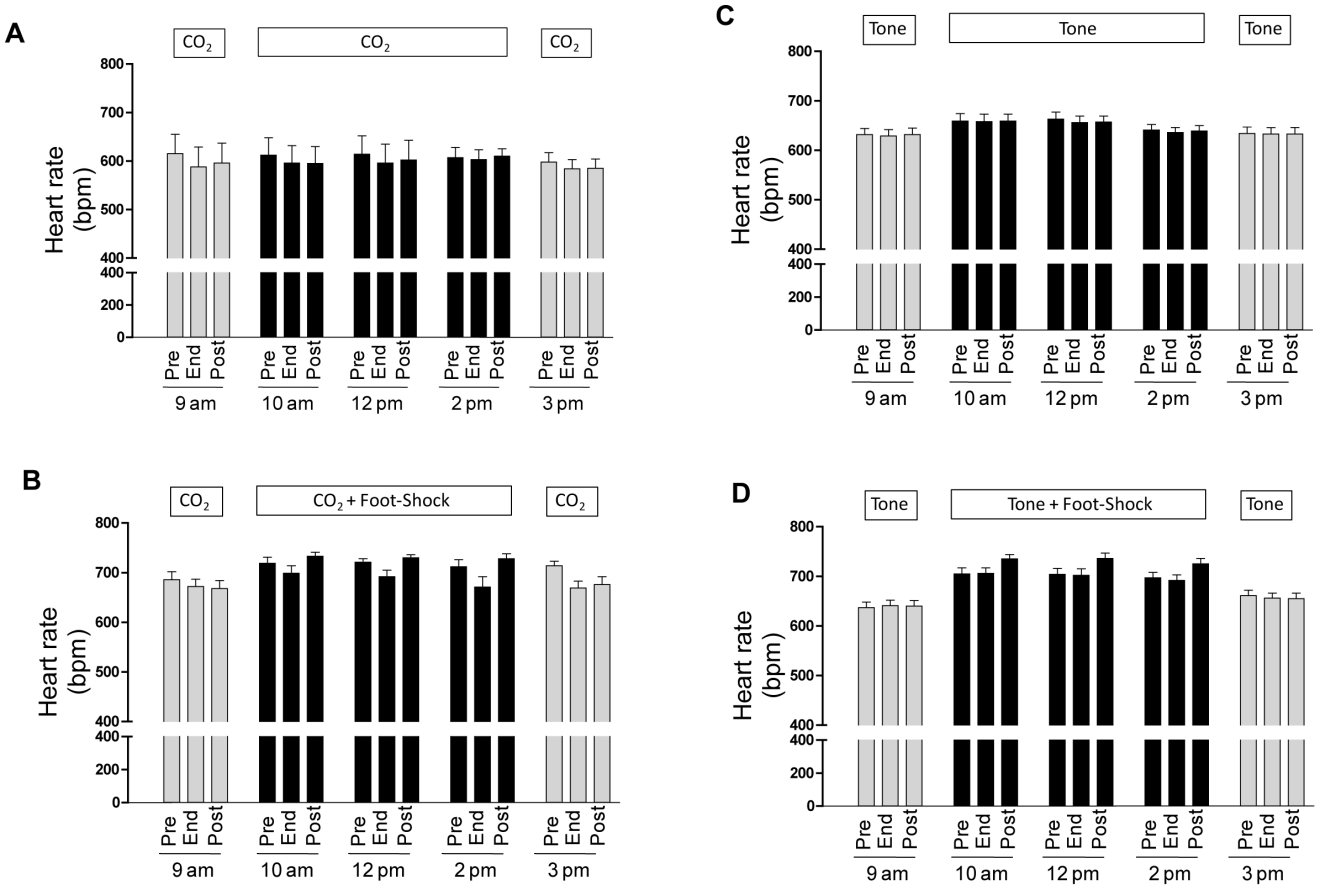
Shows the mean ± s.e.m for heart rate in beats per minute (bpm) at the Pre, End, and Post stimulus time points for each of the five training series at 9 am, 10 am, 12 pm, 2 pm, and 3 pm for the (A) the CO_2_+ footshock group, (B) CO_2_ alone group, (C) the tone+footshock group, and (D) the tone alone group.

**Figure 4:**
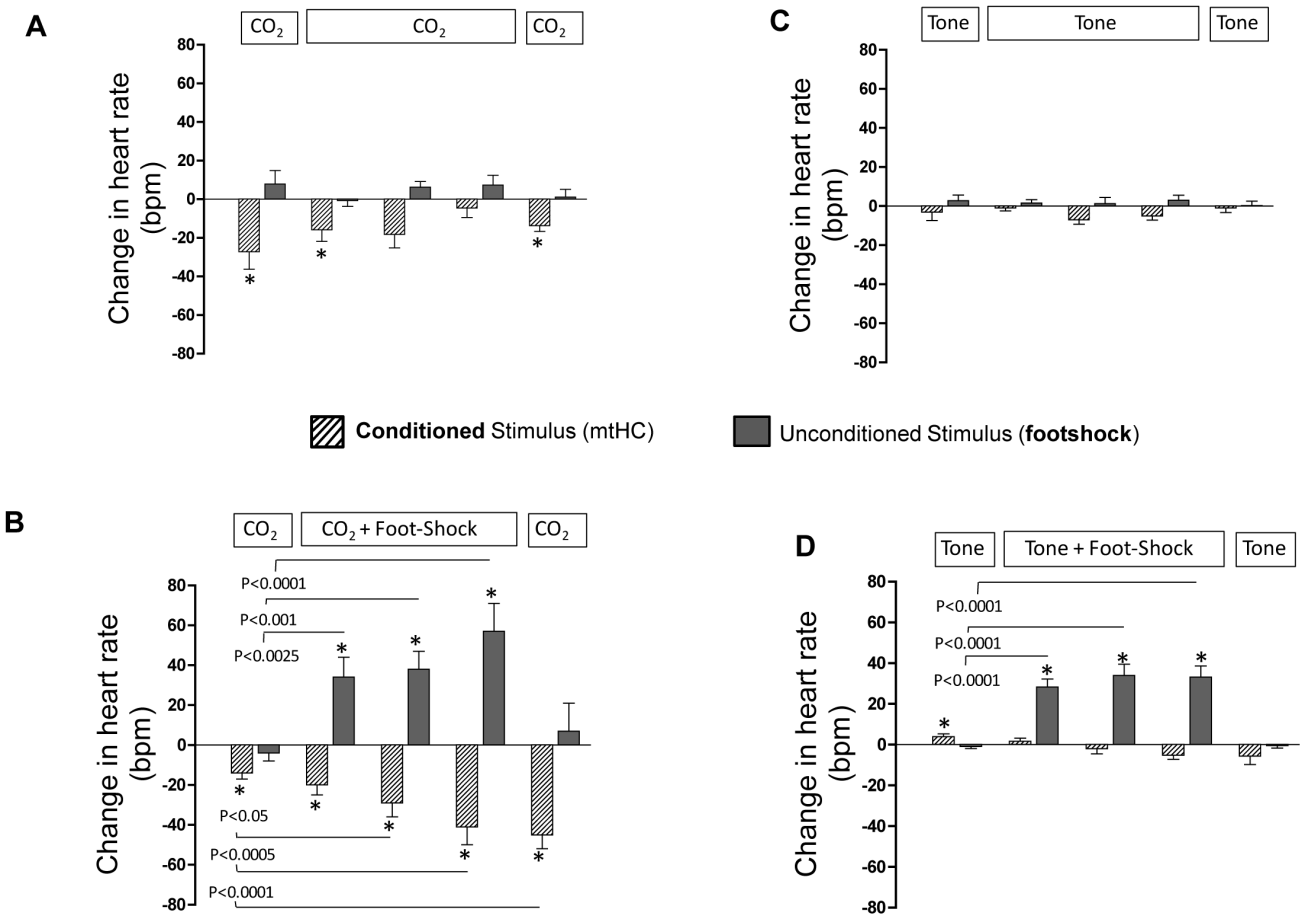
Shows the mean ± s.e.m change in heart rate in response to the CS (end-stimulus – pre-stimulus heart rate; crosshatched bars) and the US (post-stimulus – end-stimulus heart rate; dark bars). Statistical differences in heart rate across time for either the CS or US were determined by one-way ANOVA with repeated measures using a Dunnett’s post-hoc comparison to the initial exposure period at 9 am (left crosshatched and gray bar in each of the four panels. (A) A significant bradycardia effect of the CS that increased in magnitude across exposure sets and was maintained on the last CO_2_ exposure in the absence of footshock. There was also a significant US tachycardia effect during the three CS-US paired trials. (B) An initially small but significant CS bradycardia effect that habituated across exposure sets for animals exposed to CO_2_ without footshock. (C) No effect of the CS, but a significant US tachycardia during the three CS-US paired trials in animals exposed to tone with footshock. D) Negligible CS effects for tone alone exposures. *p<0.05.

Absolute mean heart rates during the five exposure periods of fear conditioning for all four groups of animals were within the normal response range for conscious, chronically instrumented mice (Figure 3). Figure 4 shows the changes in HR during the presentation of the CS, as well as the changes occurring post US for all four groups. There was an overall significant effect of group during CS presentation (F = 25.36, p<0.01) and US presentation (F = 18.75, p<0.001). When mtHC was paired with footshock it induced a marked bradycardia that increased in magnitude across series demonstrating a learned response sensitization (Figure 4A, three middle crosshatched bars), with a mean value of −31±4 bpm and a maximum value above 40 bpm (see also heart rate tracing in Figure 2 as an example of a large bradycardic response). Footshock induced a tachycardic response with a mean response of 44±7 bpm (Figure 4A, three middle black bars). Notably, the magnitude of the bradycardia was sustained (>40 bpm) in the final CS series of exposures in the absence of footshock (Figure 4A, far right crosshatched bar), whereas the tachycardia response was not (Figure 4A, far right black bar).

A different heart rate response pattern was observed when mtHC was presented alone. The initial mtHC exposure in the unpaired (Figure 4B) group was similar to the paired group’s initial mtHC alone series with a response pattern of mild bradycardia to CO_2_ and no tachycardia in the absence of footshock. For the unpaired group there was a relatively small and inconsistent mean bradycardic response of −13±4 bpm (Figure 4B, three middle crosshatched bars) that did not show a learned response sensitization and was found to be significantly different from the paired mtHC group in the post hoc analysis (p<0.01). As expected there was no tachycardia generated in the absence of footshock which contrasted with the significant tachycardia exhibited in the paired mtHC group (p<0.01) (Figure 4B vs. 4A, black bars). Thus, the bradycardia occurring during paired mtHC and footshock represents a learned physiological response to a novel conditioned stimulus, which did not extinguish during the fifth exposure series of mtHC alone.

In contrast to what was seen with mtHC, when tone was paired with footshock there was no learned heart rate response to tone alone (p>0.05) and a negligible mean change (−1±1 bpm); however, there was the expected tachycardic response to footshock of 31±3 bpm (Figure 4C). For the tone alone group there were also negligible changes in mean heart rate across the repeated exposures of −4±1 bpm and no tachycardic response in the absence of footshock (Figure 4D).

#### Mean arterial blood pressure

**Figure 5:**
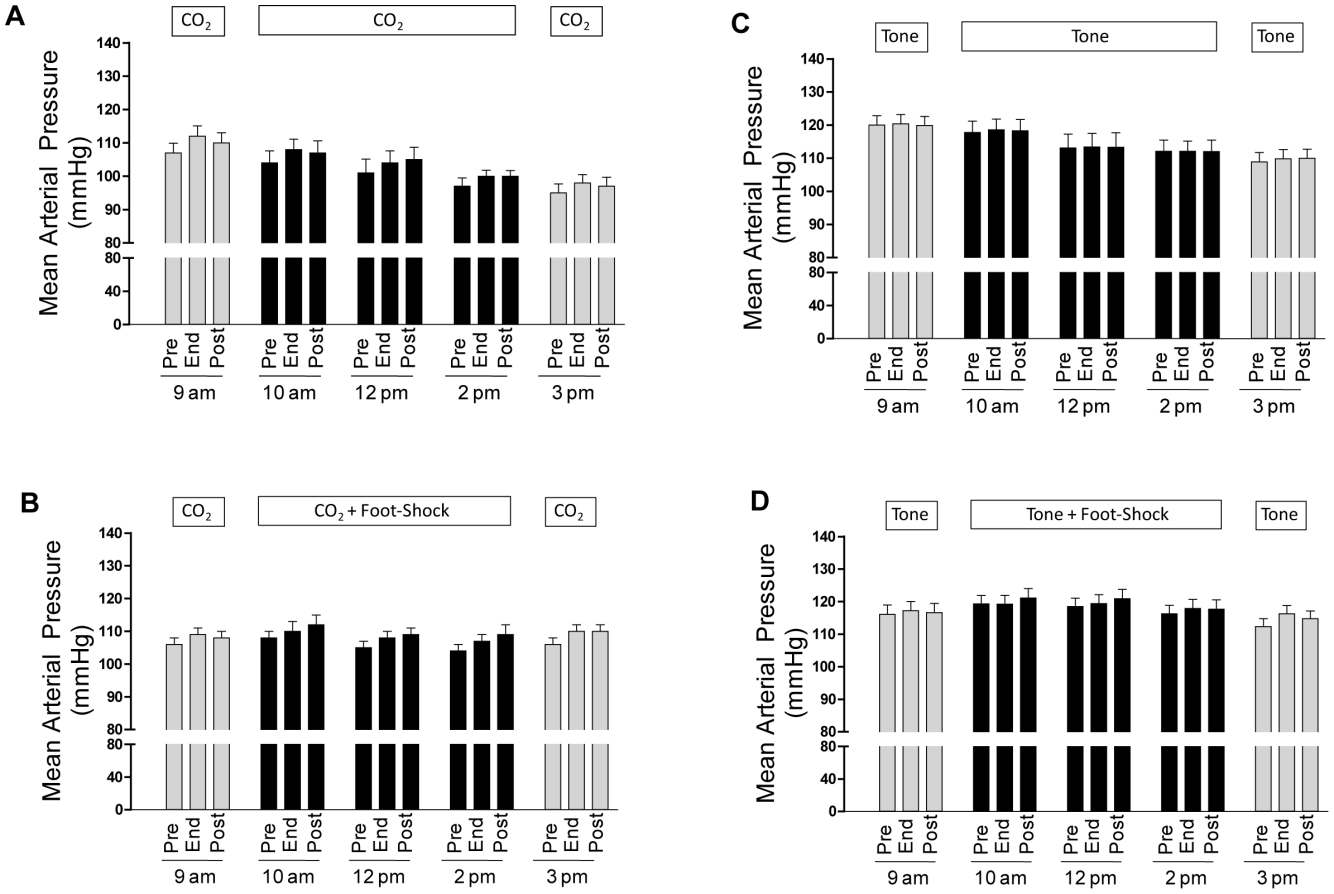
Shows the mean ± s.e.m for mean arterial pressure at the Pre-, End-, and Post-stimulus time points for each of the five training series at 9 am, 10 am, 12 pm, 2 pm, and 3 pm for the (A) the CO_2_+ footshock group, (B) CO_2_ alone group, (C) the tone+footshock group, and (D) the tone alone group.

**Figure 6:**
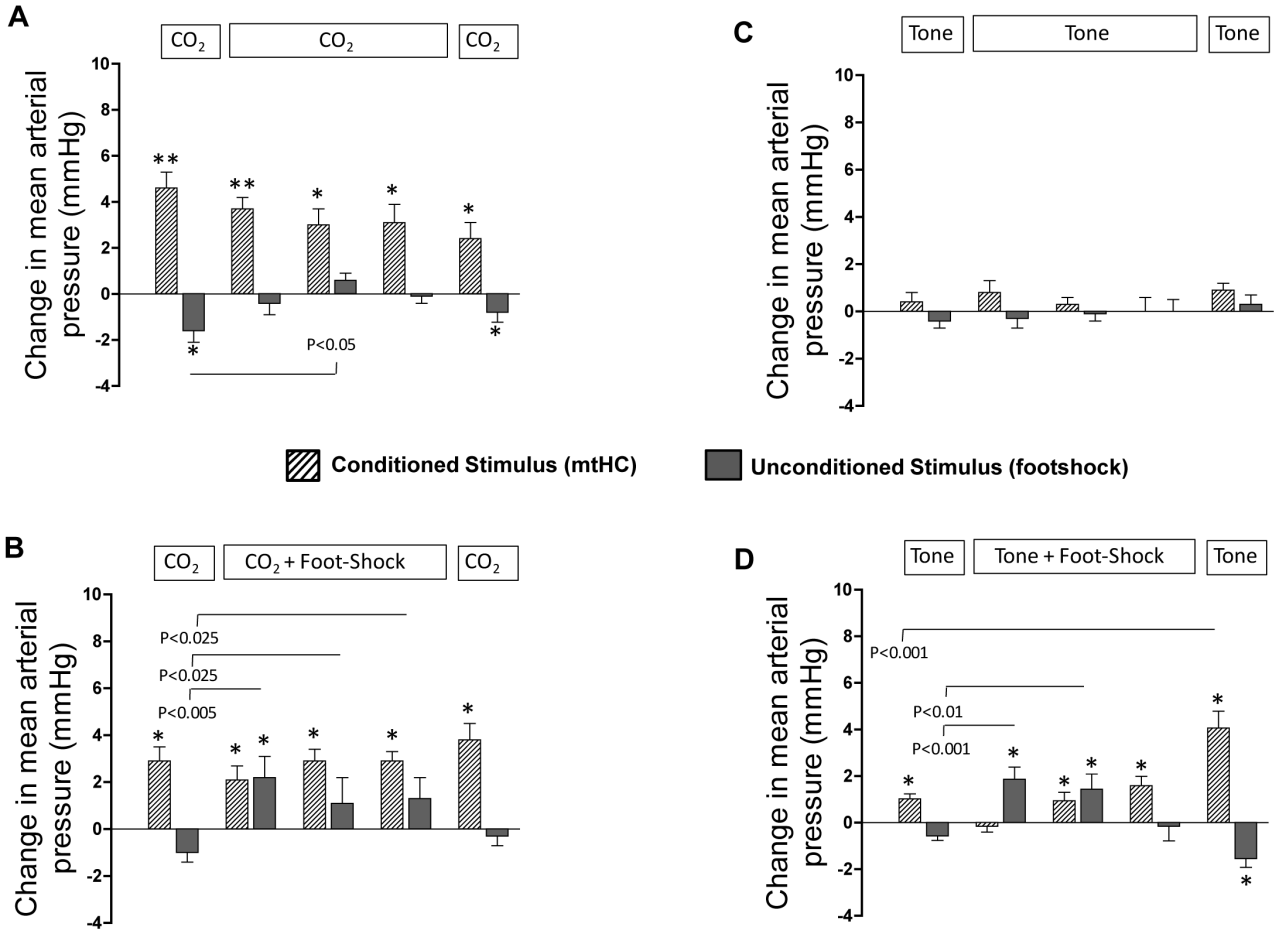
Shows the mean ± s.e.m change in mean arterial pressure in response to the CS (end-stimulus minus pre-stimulus mean arterial pressure; crosshatched bars) and the US (post-stimulus minus end-stimulus mean arterial pressure; dark bars). Statistical differences in mean arterial pressure across time for either the CS or US were determined by one-way ANOVA with repeated measures using a Dunnett’s post-hoc comparison to the initial exposure period at 9 am (left hand crosshatched and gray bar in each of the four panels. (A) The initial mtHC alone series produced a small but significant hypertensive response as did exposure to the three US periods of footshock. (B) A small but significant CS hypertensive response across all exposure sets for animals exposed to CO_2_ without footshock. (C) Arterial blood pressure responses to the tone paired with footshock produced a small, but significant increase in mean arterial blood pressure in two of the first three paired exposures. (D) Responses to tone alone were small, inconsistent and had no effect on mean arterial blood pressure. *p<0.05.

Absolute mean arterial blood pressures were also in the normal range for conscious, chronically instrumented mice during the five exposure periods for all four groups of animals (Figure. 5). Exposure to mtHC alone caused a small increase in blood pressure that was consistent across all exposures and was independent of whether it was paired with footshock (mean response of 3.0±0.3 mmHg) or unpaired (mean response of 3.0±0.4 mmHg; p>0.05; Figure 6A and 6B, crosshatched bars).

The effect of tone on blood pressure was small and relatively inconsistent (Figure 6C and 6D, crosshatched bars) and unrelated to whether it was paired (mean response of 0.8±0.2 mmHg) or unpaired (mean response 0.4±0.2 mmHg) with footshock (p>0.05; Figure 6C and 6D). A small increase in blood pressure occurred across the three paired CS-US periods and was not different between the mtHC and tone stimuli (Figure 6A and 6C). However, comparing all series between groups, the small but consistent hypertensive response to mtHC seen in the presence or absence of footshock was statistically greater than for either of the two groups exposed to tone as a CS (F = 21.69, p<0.001).

Considering the blood pressure and heart rate data together we show that tone alone has no effect on heart rate or blood pressure across repeated exposures, whereas mtHC induces a mild hypertensive response and is associated with a small bradycardia. The effect of footshock induced an acute tachycardic and mild hypertensive response. Only when mtHC predicted footshock did a learned FC response develop consisting of an increasing bradycardic response across exposures that occurred in the presence of a consistent, but mild, hypertensive response which did not change across series.

#### Catecholamines

**Figure 7:**
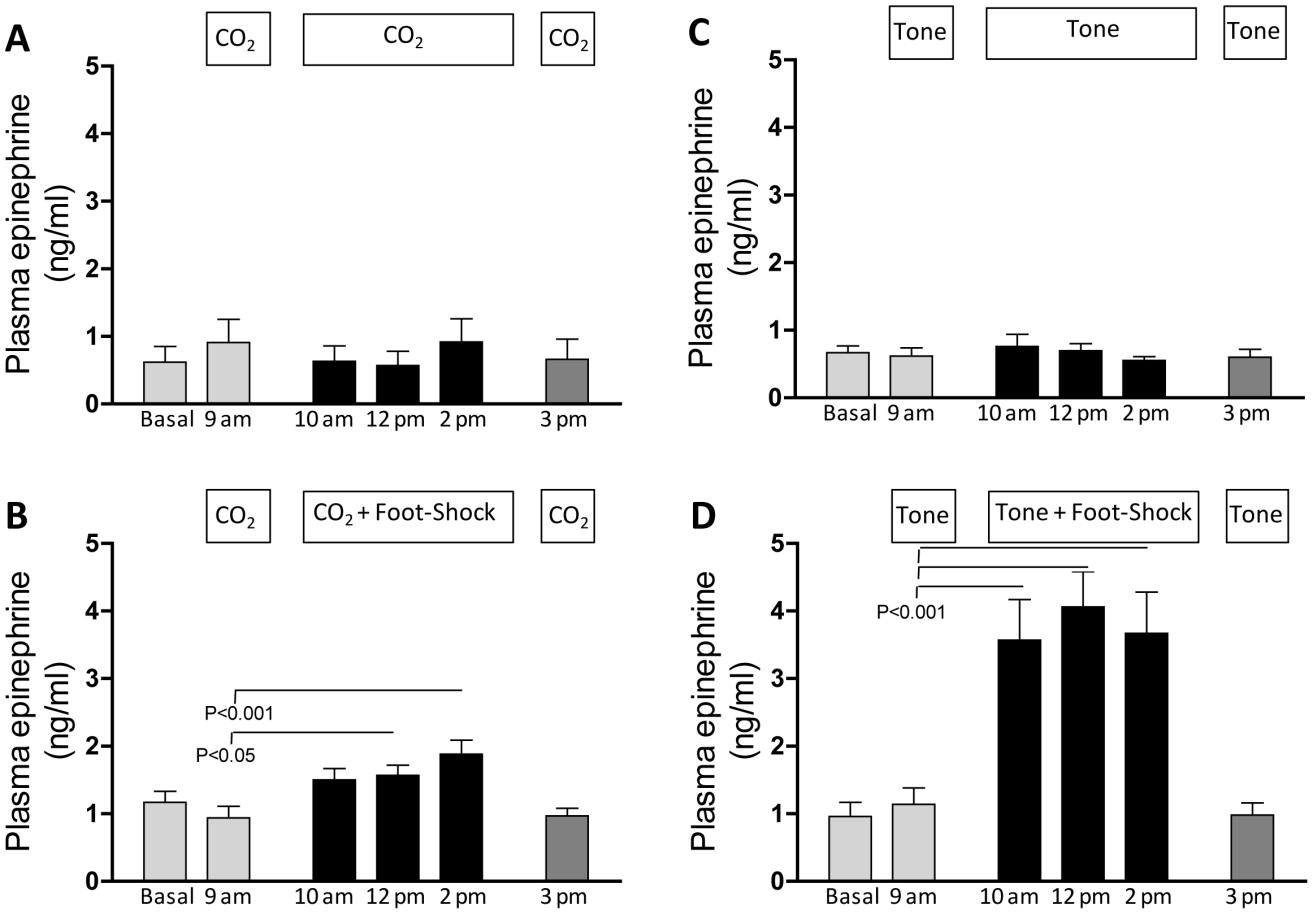
Shows the mean ± s.e.m plasma epinephrine under basal conditions and after each series of exposures to the CS or the paired CS-US at 9 am, 10 am, 12 noon, 2 pm, and 3 pm. Differences in plasma epinephrine across time were determined by repeated measures one-way ANOVA with Dunnett’s post-hoc comparisons relative to the 9 am presentation of the conditioned stimulus alone.

There was a small, but statistically significant increase in plasma epinephrine during the last two presentations of the paired CS-US for the mtHC+FS exposure (Figure 7A, two right black bars). There was a return of plasma epinephrine to baseline levels for the re-exposure to mtHC alone series at 3 pm (Figure 7A, far right dark gray bar). We did not see the same response pattern for the mtHC alone (control) group, with plasma epinephrine remaining at basal levels across all six samples taken (Figure 7B). The T+FS group exhibited a three to four-fold increase in plasma epinephrine and similar to the paired mtHC group plasma epinephrine returned to baseline levels after the final series of the tone alone (Figure 7C, right dark gray bar). Consistent with the mtHC alone group, there was no change in the response pattern across all six series for the tone alone group (Figure 7D). When comparing between groups, there was an overall significant effect of group (F = 41.74, p<0.001) and post hoc tests revealed that plasma epinephrine in response to the three pairings of CS-US was significantly higher in the tone+FS group than the mtHC+FS group (p<0.01), and both were significantly higher than their non-shocked counterparts (p<0.05).

#### Sleep

**Figure 8:**
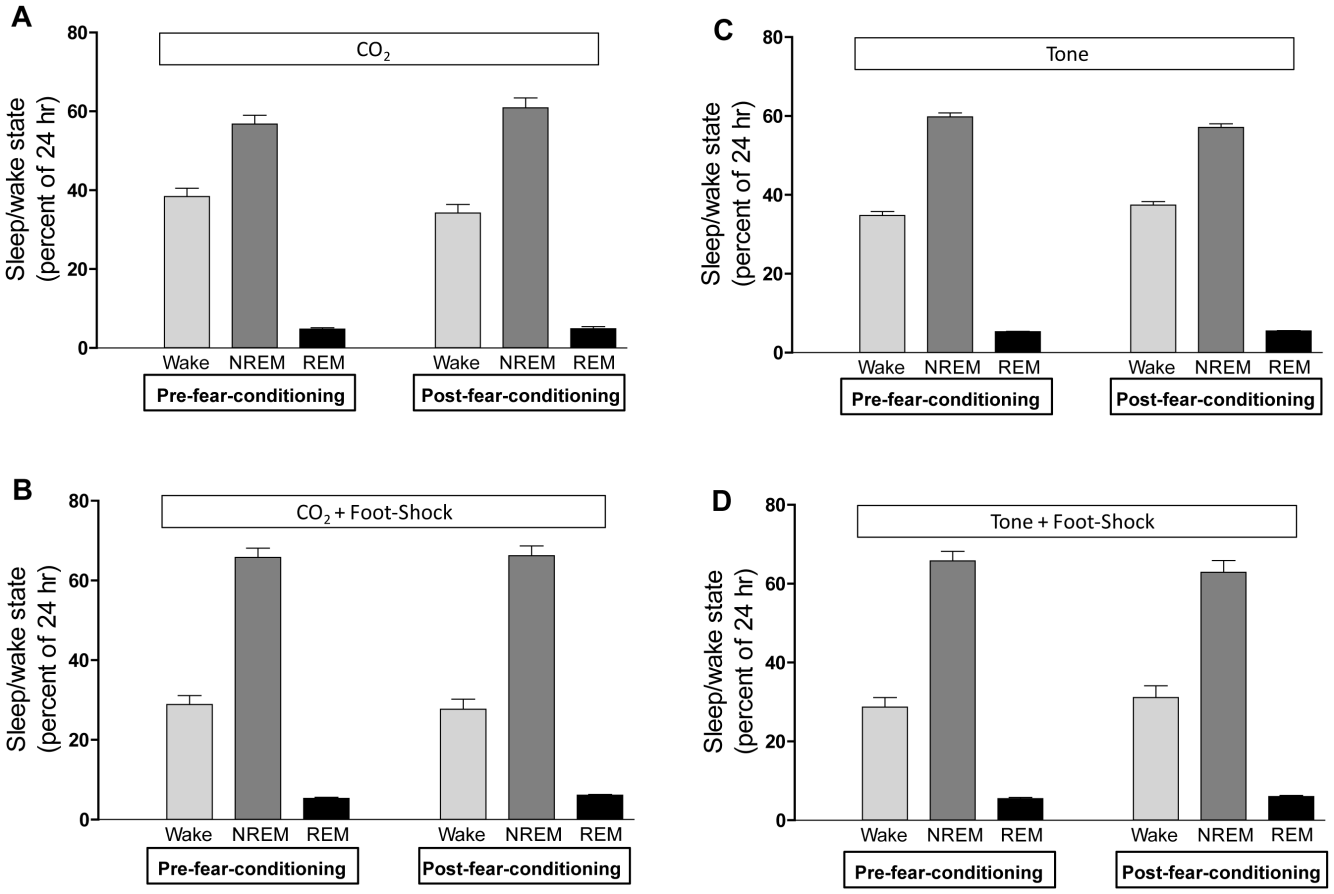
Shows the percent of time spent in wakefulness, NREM sleep, and REM sleep in the 24 hour period immediately post-fear-conditioning was compared to the same 24 hour period on the day prior to fear-conditioning for each group. Differences in time spent in each sleep state between pre- and post-fear conditioning were assessed by paired Student’s t-test. There were no significant differences in any of the four groups before fear conditioning compared to after fear conditioning for any sleep/wake state.

The percent of time spent in wakefulness, NREM sleep, and REM sleep during the day prior to FC and the day after FC is shown in Figure 8. There were no differences in total time spent in any of the three sleep/wake states within or between groups on either the pre-FC day or the post-FC day.

### Experiment 2

#### Heart rate

**Figure 9:**
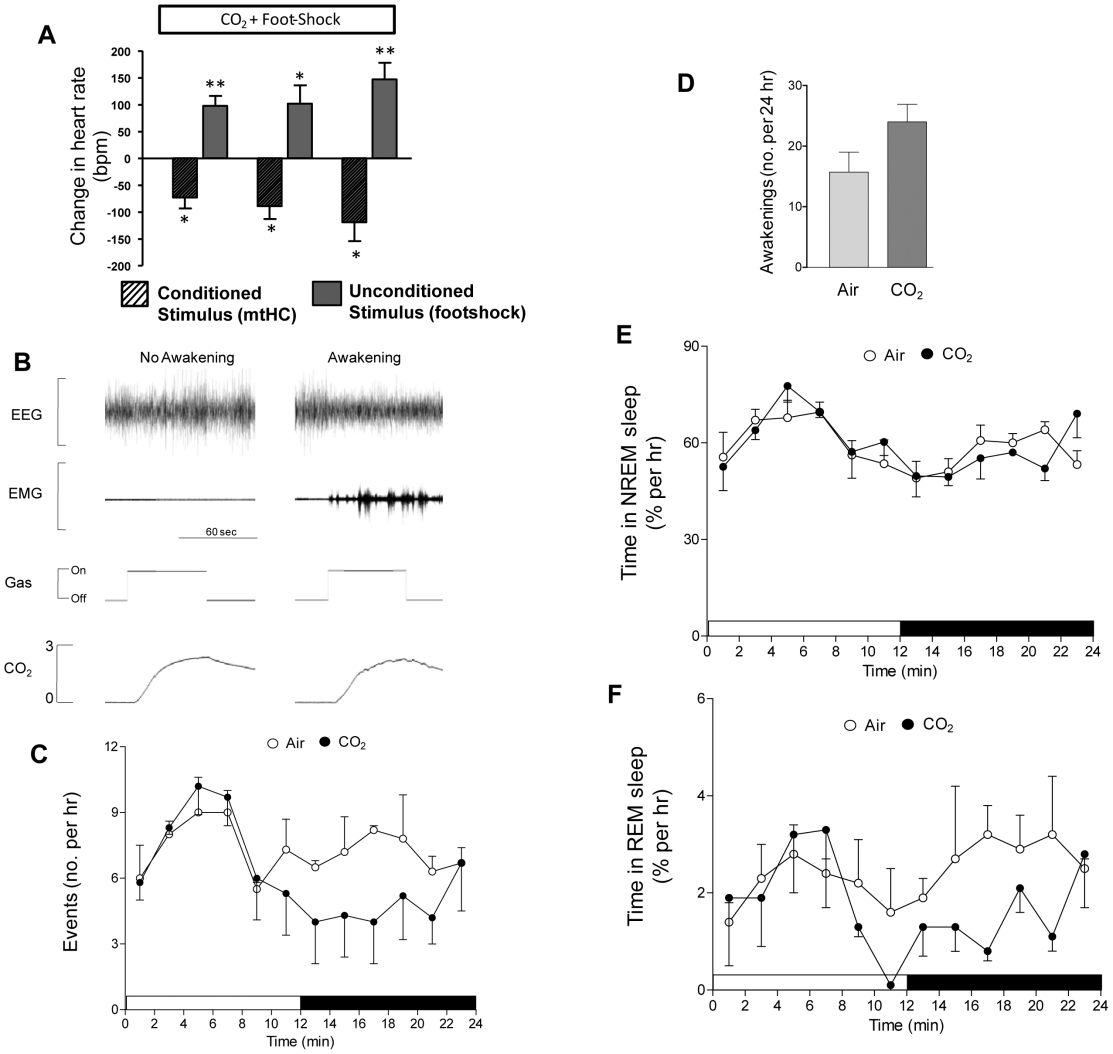
Shows a proof of principle pilot study demonstrating the utility and impact of re-exposure to a CS of 3% CO_2_ for 60 sec whenever three minutes of consolidated sleep occurred. (A) Demonstration of the learned bradycardic response to the CS of 3% CO_2_ (similar to that shown in Figure 4B) that increased in magnitude across exposures; also, the US of footshock produced the expected tachycardic response. (B) Two sample tracings from a mouse showing a 60 sec exposure to 3% CO_2_ during sleep with one event having no impact on sleep (left) and the other causing a distinct awakening (right). (C) The number of gas exposure events was averaged in two hour bins across the 24 hour re-exposure period for animals that were re-exposed to the CS+ (3% CO_2_) compared to those re-exposed to the CS− (air). (D) The total number of awakenings across the 24 hour period after fear conditioning for animals re-exposed to the CS+ and to the CS−. (E) Time in REM sleep was averaged in two hour bins across the 24 hour re-exposure period for animals that were re-exposed to the CS+ and to the CS−. (F) Time in NREM sleep was averaged in two hour bins across the 24 hour re-exposure period for animals that were re-exposed to the CS+ and to the CS−.

We assessed change in heart rate in response to the three exposures of paired CS-US in the six mice that underwent FC in Experiment 2 (Figure 9A). We reproduced a similar pattern of marked bradycardia in response to mtHC preceding footshock as seen in Experiment 1 (three middle crosshatched bars in Figure 4A). However, the degree of bradycardia we saw in Experiment 2 (without prior exposure to mtHC alone) reached a magnitude of −118±34 bpm during the third exposure period (Figure 9A, far right crosshatched bar). In contrast, in Experiment 1 when animals experienced prior exposure to mtHC alone before the repeat CS-US pairings the comparable bradycardia was only −41±9 bpm (Figure 4B, second crosshatched bar from right).

#### Re-exposure to mtHC during sleep

Sample tracings in Figure 9B show two separate one-minute periods of mtHC exposure triggered automatically after three minutes of continuous sleep in one mouse. In the first sample trace mtHC had no impact on sleep state whereas the second period of mtHC induced an awakening. We show the mean number of events, percent time in NREM sleep and percent time in REM sleep across the 24 hr re-exposure period in Figure 9C, 9E and 9F.

## Discussion

The primary purpose of the current study was to develop and test a novel conditioning stimulus for the purpose of re-exposing the animals during sleep as a proof of concept to study fear conditioned processes during sleep. We determined that mtHC produced a robust, reproducible learned bradycardia response when paired with footshock that was not seen with mtHC alone or tone+footshock. Assessment of systemic stress through measurement of circulating epinephrine demonstrated an absence of response to mtHC alone and when mtHC was paired with footshock the increase in epinephrine was less than seen with the traditional pairing of tone+footshock. We subsequently demonstrate in a proof of principle study that mtHC can be reapplied during sleep in FC animals allowing a new experimental paradigm for future studies to examine unique relationships between learning, memory, and sleep with potential application to the field of PTSD.

Our primary goal was to establish mtHC as an acceptable CS and in doing so we compared the physiological responses of our model with the responses to the more traditional FC stimulus of tone. Experiment 1 involved repeated CS-US exposures and included exposure to the CS alone both before and after the three periods of CS-US pairings. We performed the repeated exposures of CS and CS-US to verify that any cardiovascular changes we observed were physiologically meaningful and reproducible. Exposure to the CS alone before and after the three periods of paired CS-US was designed to allow each animal to act as its own control and determine whether specific cardiovascular response patterns were acquired. We chose to study the FVB/J strain because our previous work characterized this strain as exhibiting ‘hyperadrenergic’ cardiovascular responsiveness [34], [35]. We were, therefore, surprised to observe that the unconditioned footshock stimulus produced relatively small increases in blood pressure (1–2 mmHg) associated with an acute tachycardia of only 30–40 bpm. These relatively minor and transient cardiovascular changes and the lack of sustained plasma epinephrine levels in the final exposure series suggest that the tachycardic responses to footshock exposures resulted in only an acute disruption of physiologic homeostasis. Even though we observed a robust learned bradycardia in response to our FC paradigm, we did not see any changes in sleep architecture in the subsequent 24 hour period. Others have reported that FC with a tone-footshock paradigm can alter sleep architecture [24], [36], [37], suggesting that our specific FC paradigm (animals underwent a single day of training and were exposed to the CS alone at the end of the training session to establish that the bradycardic response persisted in the absence of pairing with footshock) or strain choice (FVB/J) may have mitigated perturbations in sleep. Nevertheless, the marked bradycardic responses we report demonstrate that mtHC is a viable CS with the potential for re-exposure during sleep.

We chose 3.0% CO_2_ as a novel conditioning stimulus because we know that it is sensed by chemoreceptors during sleep, but is sufficiently mild to elicit minimal cardiorespiratory effects [26]. Our data show that 60 sec exposure to 3.0% CO_2,_ in the absence of footshock, produces a 2–3 mmHg increase in mean arterial pressure associated with an initial bradycardia of ∼20 bpm, which habituated across series (see Figure 4B). When mtHC predicted footshock there was an acquired bradycardia response that increased in magnitude with successive series of FC and was maintained in the final series with representation of the CS alone even though the tachycardic US response was not. This response pattern was not seen in the mtHC alone group, which demonstrates that the effect cannot be solely due to mtHC alone nor can it be due to footshock. Therefore, we suggest that mtHC is an effective CS that can induce associative conditioning, while possessing appropriate physical properties for redelivery during sleep.

There was also a small, but significant increase in plasma epinephrine for the paired mtHC group and a large increase for the paired tone group. Both groups returned to baseline in response to the final presentation of the CS alone suggesting that the catecholamine effects seen were primarily due to the acute responsiveness to footshock, but given the difference in magnitude this suggests that the strength of the CS-US pairing also plays a role in the acute epinephrine response. Overall, our basal plasma epinephrine levels were approximately three times lower than previously reported in anesthetized mice [38], which is consistent with our ability to sample blood from unhandled and unstressed animals. Thus, mtHC when paired with footshock elicits a reduced systemic stress response relative to tone paired with footshock, providing further support that it is a CS with the potential for re-exposure during sleep.

In Experiment 2, where mtHC was not presented alone before the CS-US pairings, the magnitude of the bradycardic response was approximately three times greater than in Experiment 1 where mtHC was presented alone before the CS-US pairings. Therefore, prior exposure to the mtHC partially inhibited the subsequent learned bradycardic response to the paired CS-US exposures, which suggests a latent inhibition effect [39]. We acknowledge that alternative experimental designs involving a smaller number of CS-US exposures may be more appropriate in studies involved in dissecting specific aspects of learning and memory, particularly those utilizing tone as a conditioned stimulus. However, for our purposes multiple pairings were appropriate for this study to examine the within subject physiological responsiveness across exposure periods to assess individual pre- and post-stressor effects.

Hyerpcapnia stimulates central and peripheral chemoreceptor pathways and activates multiple brainstem neuronal centers [40]–[43]. Neural connections between the brainstem and amygdala, which is an area that is necessary and sufficient to produce cue-specific learning and memory of fear responses [12], [14], [44], likely contribute to the CS-induced bradycardia we report with mtHC. Additionally, mtHC may also directly activate pH sensitive acid sensing ion channel-1a (ASIC1a) that detects CO_2_ within the amygdala [45]. It is important to note, however, that in this study by Ziemann and colleagues [45] they found evidence of FC only at extremely high levels of 10% CO_2_, whereas our observation of learned bradycardia was evident with transient (60 sec) and very mild (3.0%) CO_2_. The mechanism(s) of our learned bradycardia are not clearly understood at this point, but it is possible that repeated pairings may activate ASIC channels within the basolateral amygdala and through alteration of the membrane potential [46], [47] increase the likelihood of coincidence detection to account for the conditioned bradycardia effect. Thus, mtHC constitutes a unique CS that may directly (ASIC channels) and indirectly (projections from the brainstem) activate the amygdala to induce a learned bradycardic response when paired with footshock.

The other primary goal of our study was to develop a CS for re-exposure during sleep. We piloted the principle of mtHC exposure during sleep and compared outcomes with an air control stimulus to account for any non-specific effects of gas flow changes. Although our number of subjects limited our ability to test for statistical differences, it will be interesting in future studies to determine if re-exposure to mtHC can increase the total number of awakenings and potentially lead to deficits in REM sleep. The application of mtHC re-exposure during sleep may be used to explore the impact of genetic strain, specific candidate genes, neurodevelopment and other important clinical correlates on the relationship between CS and sleep. The sleep re-exposure model may also be applied to other aspects of learning and memory. For example, does re-exposure to a previously fear conditioned CS during sleep impact learning a novel task associated with the CS during acquisition or consolidation (e.g., increased discrimination learning)? Alternatively, could CS re-exposure during sleep or wakefulness be used as a tool to hasten extinction of a traumatic CS-US pairing as could occur in military environments in patients with PTSD? For example, re-exposure to the CS may impact extinction renewal processes and reconsolidation. To our knowledge mtHC has not been used as a CS in human studies. However, much higher levels of acute exposure to CO_2_ (e.g., 35%) have been used to induce panic attacks in humans [48] (note: the much lower level of 3% CO_2_ that we have used in mice did not elicit panic based on direct observation, as well as heart rate and blood pressure responses). Potentially, 3% CO_2_ could be used as a CS in human sleep studies since the increase in arterial pCO_2_ [49] is below the arousal threshold [50]. Probing sleep-specific events during re-exposure to the CS may address disrupted sleep patterns that are known clinical correlates in veterans with PTSD [2], [51]. Our current model provides a new avenue to study fear-induced activity during sleep and to answer these important questions under controlled laboratory conditions.
